# Data Gathering and Energy Transfer Dilemma in UAV-Assisted Flying Access Network for IoT

**DOI:** 10.3390/s18051519

**Published:** 2018-05-11

**Authors:** Sara Arabi, Essaid Sabir, Halima Elbiaze, Mohamed Sadik

**Affiliations:** 1 NEST Research Group, Laboratoire de Recherche en Ingénierie (LRI), ENSEM, Hassan II University of Casablanca, Casablanca 8118, Morocco; sara.arabi@ensem.ac.ma (S.A.); m.sadik@ensem.ac.ma (M.S.); 2 Computer Science Department Research Group, University of Quebec at Montreal (UQAM), Montreal, QC H2L 2C4, Canada; elbiaze.halima@uqam.ca

**Keywords:** unmanned aerial vehicle (UAV), Internet-of-Things, energy harvesting, data collection, wireless recharging, scheduling time, UAV trajectory planning

## Abstract

Recently, Unmanned Aerial Vehicles (UAVs) have emerged as an alternative solution to assist wireless networks, thanks to numerous advantages they offer in comparison to terrestrial fixed base stations. For instance, a UAV can be used to embed a flying base station providing an on-demand nomadic access to network services. A UAV can also be used to wirelessly recharge out-of-battery ground devices. In this paper, we aim to deal with both data collection and recharging depleted ground Internet-of-Things (IoT) devices through a UAV station used as a flying base station. To extend the network lifetime, we present a novel use of UAV with energy harvesting module and wireless recharging capabilities. However, the UAV is used as an energy source to empower depleted IoT devices. On one hand, the UAV charges depleted ground IoT devices under three policies: (1) low-battery first scheme; (2) high-battery first scheme; and (3) random scheme. On the other hand, the UAV station collects data from IoT devices that have sufficient energy to transmit their packets, and in the same phase, the UAV exploits the Radio Frequency (RF) signals transmitted by IoT devices to extract and harvest energy. Furthermore, and as the UAV station has a limited coverage time due to its energy constraints, we propose and investigate an efficient trade-off between ground users recharging time and data gathering time. Furthermore, we suggest to control and optimize the UAV trajectory in order to complete its travel within a minimum time, while minimizing the energy spent and/or enhancing the network lifetime. Extensive numerical results and simulations show how the system behaves under different scenarios and using various metrics in which we examine the added value of UAV with energy harvesting module.

## 1. Introduction

Over the past few years, Unmanned Aerial Vehicles (UAVs), also known as drones, have gained tremendous attention in different domains. They are particularly used in military monitoring tasks and also recently in the civilian domain such as in goods delivery (In 2013, Amazon announced submitting some 64 patents describing a UAV-based delivery system designed to safely get packages to customers in less than 30 min [1]). The interest of UAVs in the area of telecommunications has also grown exponentially. This is mainly promoted by UAV’s ability of rapid deployment and effective relocation to provide ubiquitous connectivity during temporary events with massive audience (sport events, festivals, or congresses). UAVs can also replace damaged communication infrastructure when a natural disaster happens (earthquake, volcano, etc.). In contrast, terrestrial infrastructures are unadapted to emergencies due to their limited mobility, along with their high cost and deployment time (see [2]). There are two types of UAVs: The first one, called High Altitude Platforms (HAP), (Google uses stratospheric balloons to provide Internet access for uncovered zones [3]. Facebook Aquila is also building an alternative solution using this time flying wings with a 42 m wingspan), can reach high altitudes that can exceed 10 km, and Low Altitude Platforms (LAP) that operate at lower altitudes. In telecommunications, LAP can cover geographical areas with high capacity and better energy/spectrum utilization. Meanwhile, the HAP can provide broadband coverage and can also serve as very low altitude satellites. Thus, low altitude UAVs, also named as drone small cells (DSC), can operate as a flying base station to spread out the communication coverage for ground mobile users, and to improve user service. This kind of UAVs can also carry out maintenance services or capture information from unreachable places. This way, UAVs can be efficiently used to gather data from ground/underwater sensors. We believe UAVs are about to become a corner piece of the rushing new Internet of Things (IoT) era that is forming the future for mankind.

The increasing number of connected devices has resulted in an ultra-dense new network paradigm, called IoT. According to Gartner (https://www.gartner.com/newsroom/id/3598917), the number of connected objects/things will hit 20.4 billion by 2020. IoT combines simple objects like sensors, and more complex systems such as industrial equipment, connected/autonomous vehicles, smart buildings, etc., which exchanges with the cloud and talk to each other in order to propose new value-added services. Moreover, IoT devices must be able to collect, store, transfer and process the related data without interruption between the physical and virtual worlds [4,5]. However, like other promising concepts, this paradigm copes with a number of issues that need to be studied, enabling the IoT paradigm to reach its full potential. In addition, the incessant demand for higher data rates with better Quality of Service (QoS) requires continuous development in order to emerge. A key feature enabling achieving this is how to allow devices to use their energy budget efficiently. In other words, one needs to build solutions aiming to boost up their endurance in terms of energy. This point will be discussed in this work. Of course, the energy constraint is critical for effective self-organization, and this is necessary in order to meet communications with satisfactory QoS. Thus, it is necessary to address how IoT devices might effectively use their energy budget and extend thereby their battery lifetime. In this respect, there are a lot of works that have already studied the energy efficiency issue for IoT contexts. Nevertheless, existing solutions for continuously or on-demand supply devices with energy are very limited and the issue is still open. The question that arises is how to recharge IoT devices wirelessly and remotely. For this purpose and through this work, we propose a novel approach using flying base stations for energy harvesting on downlink to ground IoT devices in order to maintain their availability and basic operations. Before studying and analyzing the present model, we briefly remind the motivated points behind claiming UAV as a central part of the future IoT ecosystems.

Why are UAVs the future of IoT? As our daily life is surrounded by thousands of connected objects: smartphones, houses, cars, smart-meters, trees, etc., forming an IoT environment, from very small scales to massive ones. They interact in many aspects of the human activities. Thus, one need accurate models to capture the objects’ behaviors around us. For this reason, the UAVs can play an important role in connecting IoT islands to the the whole IoT ecosystem. However, UAVs offer many new and flexible features such as high mobility providing ubiquitous network access and coverage for ground users and IoT devices. Their plug-and-play like deployment, their reprogrammability during run-time and their capability to capture minute details and fresh measurements are of great interest. Another reason to adopt such a solution is the energy constraint of IoT devices that needs to be fixed. Reducing the energy consumption and extending the network lifetime is a very challenging issue that has a huge impact on Ultra Dense IoT Networks, which consists of massive spatially distributed wireless nodes where batteries are the primary energy sources. Thus, and as regularly recharging or periodically replacing batteries for massive nodes can be costly and inconvenient. Here, we propose to solve this problem by introducing an UAV station for wireless energy harvesting and transfer. More precisely, this work exhibits a UAV-assisted solution providing both network and energy for IoT.

### 1.1. Related Work

Recently, many researchers have spent significant efforts to the use of unmanned aerial vehicles to extend and provide wireless communication coverage anywhere, anytime. Mozaffari et al. [6] suggests using UAVs to efficiently control energy consumption during uplink data collection from ground IoT devices. In a similar perspective, Zhan et al. [7] proposes a novel design for energy-efficient data collection in UAV-enabled Wireless Sensor Networks (WSNs). The authors in this work study the mutual optimization of sensors’ wake-up schedule and UAV’s trajectory to achieve the quoted aim (reliable and energy-efficient data collection), by using a successive convex optimization technique. Koulali et al. [8] examines the problem of optimal beaconing for drone small-cells (DSC) networks using a sub-modular game perspective. The main goal is meet satisfactory network availability in terms of encounter rate while improving energy efficiency. Furthermore, Lee [9] investigates both energy efficiency and throughput that can be achieved via large-scale antenna systems scheme as a promising solution to reduce the carbon footprint emitted by a cellular base station. An interesting solution is given for the 3D placement problem of mobile base stations (MBS) embedded in UAVs, which is investigated by Lyu et al. [10]. The authors aim to reduce the cost by minimizing the number of MBSs to be deployed, while each ground terminal must remain within the communication range of at least one MBS, based on the algorithm of Spiral MBS Placement. Other works assume path-loss, as only Line-of-Sight (LoS) is considered in the air-to-ground link, to achieve an optimal dimensioning of drone base station that leads to optimal network performance (see e.g., Ref. [11,12]). In addition, Mozaffari et al. [13] investigates the low altitude platform and the downlink coverage performance of DSCs for an efficient deployment of DSCs. In the meantime, numerous frameworks suggest using UAVs as flying base stations in order to maximize the coverage probability that depends on the UAV altitude and transmit power. Ravi and Dhillon [14] derives the coverage probability of a target receiver located on the ground using a dominant interferer based approach. Along the same line, Mozaffari et al. [15] analyze the average coverage probability and the rate performance in the presence of underlay Device-to-Device (D2D) links within a UAV transmission. Beyond security aspects, Sanjab et al. [16] focuses on the security of UAV delivery systems against cyber-physical attacks, based on zero-sum game between delivering purchases through UAVs and malicious attacks. To the best of our knowledge, the deployment of UAVs has been used in different scenarios to collect information. Nevertheless, as it can be noticed after quoting these works, the models that have been considered jointly provide network access and wireless energy transfer, with energy harvesting capability still not sufficient to definitely cover/solve the UAV-IoT area. For instance, Lee and Yang [17] propose the UAV as a medium to transfer RF energy to user equipment that suffers from limited energy and cannot directly be supported by a massive Multiple-Input Multiple-Output (MIMO) data center. Aiming to maximize the amount of energy transferred to a set of ground receivers, Xu et al. [18] uses UAV as a wireless power transfer system. In addition, the authors evaluate the UAV mobility operation in order to find the optimal trajectory.

Energy harvesting is one of the most promising solutions that emerge in self-powered systems, such as the Internet of Things [19,20]. This technique aims to generate energy from external ambient energy sources such as solar power, thermal energy and wind energy. In radio environment networks, the preferred way to harvest energy is Radio Frequency (RF) sources, due to its easy implementation within existing wireless networks and the fact that it suffers from low harvesting gain. Stations equipped with energy harvesting modules are able to convert the received signal into energy to recharge out-of-battery devices. Existing literature assumes that the Radio Frequency Energy Harvesting is an attractive technology enabling improving the network lifetime of sensor devices and reducing the cost of battery replacement (see e.g., Ref. [21]).

Among several similar works, Ku et al. [22] and Ulukus et al. [23] propose theoretical models for energy harvesting in wireless communication networks. Hou et al. [24] proposes solving the problem of information asymmetry between Data Access Points (DAPs) that collect data from IoT devices and Energy Access Points (EAPs) used to transfer energy to devices, by designing the contract theory to model the incentive mechanism to motivate the EAP to (re)charge IoT devices. Energy consumption in sensing IoT was considered by Niyato et al. [25]. Here, the authors achieve an optimal sensing performance based on caching mechanism with a threshold adaptation algorithm to reduce the number of requests sent to a tagged sensor. However, in some frameworks, the energy harvesting has been applied in D2D networks. In [26], D2D communication is assumed as an underlay between machine type communication (MTC) and cellular networks. Moreover, a trade-off was proposed to reach an optimum amount of time that D2D users can spend to support MTC traffic in terms of energy, and using effectively their spectrum available by cellular users. Moreover, the security issue is an interesting subject in energy harvesting applications. In this regard, a novel learning scheme for RF energy harvesting has been proposed by Kawabata et al. [27], which allows Hybrid Access Point (HAP) to estimate a reliable value of power consumption against the problem of adversarial learning. Indeed, for the goal of detecting and recognizing suspicious persons in a crowd, Motlagh et al. [28] use an UAV-based IoT platform to benefit from computation offloading and improve the system reactivity. As it can be remarked after quoting these works, energy harvesting has been integrated in many wireless applications, but not sufficiently in UAV-IoT areas. We are confident that such an application can bring a lot to this class of networks.

### 1.2. Our Contributions

Legacy wireless networks are designed to connect together wireless devices like computers and mobile phones. However, in IoT, millions and billions of “things” are interconnected through wireless local area networks or through a cloud-Internet. Thus, a suitable maintenance policy of the batteries of those things is crucial for increased network lifetime. A promising solution to this issue is to enable energy harvesting, which allows on-demand producing and powering those device with clean energy.

Taking into account the limitations of IoT devices in terms of energy, storage and computation capabilities, we believe that UAVs are becoming a very attractive solution for providing an on-demand nomadic infrastructure for data collection and batteries recharging platform for IoT environments. In this respect, the key contributions of this work are fivefold and can be summarized as follows:

- The current work presents an efficient UAV downtime to collect data from ground IoT devices to forward it to the nearest gateway;
- This study proposes a novel framework that aims to extend the network lifetime by exploiting UAV high mobility to harvest energy from an IoT environment. In other words, we use the UAV as energy source to serve depleted IoT devices;
- We build a model to ensure an efficient trade-off between data gathering and energy providing to ground IoT devices. In this context, we can distinguish two trade-offs:
  - If we are seeking to maximize the data collection, what is the maximum time that UAV can provide to serve IoT devices?
  - If we are interested in achieving a certain battery level for IoT devices, i.e., a typical network lifetime, what is the data collection time that we must not exceed?
- Finally, as the UAV suffers from limited energy, the present work analyzes the UAV movement via the traveling salesman problem with time windows (TSPTW) to result in an optimum trajectory.

## 2. Problem Formulation

**Figure 1:**
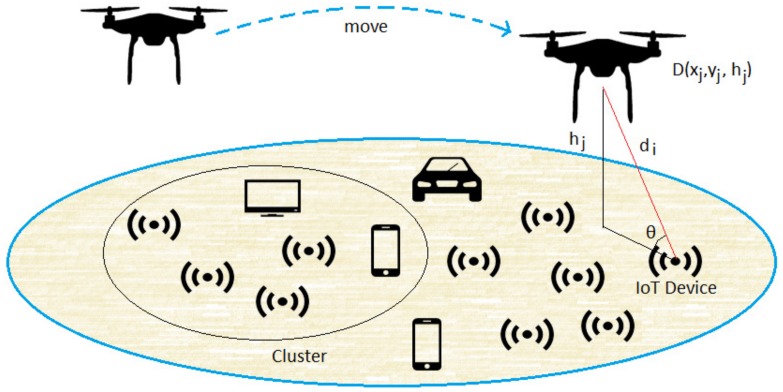
System model.

We consider a wide geographical area consisting of a set of IoT devices grouped in subsets called islands. The location of these devices follows a Poisson Point Process (PPP) in a 2D referential space (o,x,y), with some given density η of IoT device per area unit, and *o* is the origin of the coordinate system. As the IoT devices do not need to be covered all the time, we assume that the area is served and covered only by a single UAV at low altitude that act as a flying base station and equipped with an energy harvesting module. As shown in Figure 1, the UAV are coordinated randomly in a 3D reference plane (x,y,h) with altitude *h*. Throughout this paper, we try to answer the following main question: how to optimally use the coverage/hovering time for a given island to both efficiently collect data and efficiently recharge out-of-battery devices?

**Figure 2:**
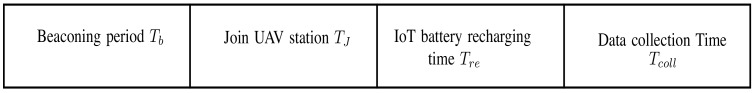
The frame structure.

Let us consider a given island N containing a set $N=\left|N\right|$ of IoT devices that are served by the UAV as a flying base station during a coverage time $T_{cover}$. The coverage time is subdivided into: synchronization time $T_{b}$, joining time $T_{J}$, energy transfer time $T_{nrg}$ and data gathering time $T_{data}$ as depicted in Figure 2. The coverage time depends on the energy available at the UAV, which gives an idea about the total time performed by UAV station to fly over all islands and hover over each one of them to transfer energy and collect data from/to ground devices, noted, $T_{tot}$. The coverage time for each island can be written as:

(1) $$T_{\mathrm{cover}}^{N}=\frac{T_{\mathrm{tot}}-T_{\mathrm{Traj}}}{N_{Net}}\cdot N,$$

where $N_{\mathrm{Net}}$ denotes the total IoT devices in the geographical area formed by all islands, and $T_{\mathrm{Traj}}$ is the time spent by the UAV station to fly over all the islands forming the network.

Before offering services provided by the UAV, namely recharging the IoT ground devices with energy and collecting data, it is essential to synchronize both the UAV station and the IoT devices in order to relocate each of them. For the lack of reducing energy consumption by ground devices, the synchronization part will be performed in a passive scanning mode. More precisely, synchronization beacons are sent to advertise the presence of the UAV station. During the joining time, each device sends its ID, its traffic rate, its battery level and potentially other information to the UAV station. This information allows the UAV station to estimate the required time for both energy transfer for low-battery devices and data gathering operation.

### 2.1. Battery (Re)charging and Data Collection Dilemma

As mentioned above, ground IoT devices will be served by the UAV embedding a flying base station. In fact, the UAV can extract energy from RF signals transmitted by IoT devices. When receiving these signals, the UAV uses a part of its power to harvest energy while the remaining part is used for data collection from devices. The coordination/attachment time between UAV station and IoT devices is shorter than both energy transfer time and data collection time. In this article, we consider the same attachment time for all the islands. Accordingly, we assume that the coverage time is split into two parts weighted by α; battery recharging time $T_{nrg}=\alpha \cdot T_{cover}$, and collection time noted $T_{data}=\left(1-\alpha \right)\cdot T_{cover}$. Subsequently, the coverage time in a given island is as follows:

(2) $$T_{cover}=T_{data}+T_{nrg}.$$

Notice that the amount of transferred energy to IoT devices is an increasing function with respect to α, while the amount of data collected is a decreasing function with α. This is why an efficient trade-off must be found in order to ensure the success of both operations (i.e., data collection and energy transfer).

### 2.2. Coverage Probability of a UAV

The UAV hovers over the islands and stops temporarily in order to collect sensed data and recharge the out-of-battery IoT devices. The duration and the location of a UAV stops are chosen for operational efficiency purpose under some constraints such as the IoT devices’ location and their respective lifetimes. UAV stops will ensure a wireless coverage allowing efficient data collection from ground IoT devices and at the same time an opportunity to recharge their low batteries. Therefore, to efficiently establish ground-to-air communication, it is essential that each device has a Line-of-Sight view toward the UAV. Taking into account the constraints already quoted and the elevation angle between the device and the UAV, the LoS probability can be written as follows [6]:

(3) $$P_{LoS}=\frac{1}{1+Ce^{-B\left(\theta -C\right)}},$$

where

- θ is the elevation angle that equals to: $\frac{180}{\pi }\cdot sin^{-1}\left(\frac{h_{j}}{d_{i,j}}\right)$;
- $d_{i,j}$ is the Cartesian distance between IoT device *i* and UAV *j* (see Figure 1). It is given by:
  $$d_{i,j}=\sqrt{{\left(x_{i}-x_{j}\right)}^{2}+{\left(y_{i}-y_{j}\right)}^{2}+h_{j}^{2}};$$
- *C* and *B* are constant values that depend on the propagation environment.

As stated earlier, ground IoT devices communicate with the UAV in a line-of-sight fashion. Moreover, we consider a one-hop routing approach and use free space channel. Thus, the expected path-loss $P_{loss}\left(h\right)$ can be written, see e.g., Ref. [12]:

(4) $$P_{loss}\left(r,h\right)={\left(h^{2}+r^{2}\right)}^{-v/2},$$

where *r* is the distance between the origin of the coordinate system and a given IoT device, and *v* is the path loss exponent depending on the environment. Thus, the coverage probability can be written as:

(5) $$P_{cover}=P\left(\frac{P_{loss}^{i}\left(r,h\right)\cdot P_{i}}{\sigma^{2}+\sum_{l\in C\setminus \{i\}}P_{loss}^{l}\left(r,h\right)\cdot P_{l}}>\gamma_{th}\right),$$

with $\sigma^{2}$ denoting the variance of an additive Gaussian noise, and $P_{i}$ is the power chosen by device *i*. $\gamma_{th}$ denotes the SINR threshold required by device *i* to correctly decode received frames.

### 2.3. Battery Level of IoT Devices: A Birth-Death Process

In order to derive the number of devices with a low battery level, we model the battery state B(i) of IoT device *i* by a Birth-Death process. The battery level is assumed to be discrete and takes value in $\left\{B_{0},B_{1},\cdots ,B_{M}\right\}$. Furthermore, and so as to capture the need for the energy of an IoT device, we define an upper battery level threshold $\bar{B}$, indicating the need to recharge battery when there is an opportunity. We also set a lower battery level threshold noted $\underline{B}$. This latter parameter indicates that the IoT devices are unable to transmit data, as their battery levels are critical and need to be recharged as soon as possible. In order to avoid IoT device from switching off, we need to adopt a smart energy transfer policy.

**Figure 3:**
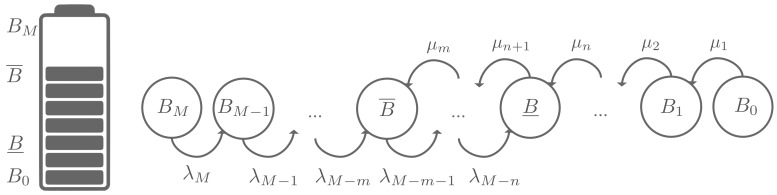
IoT device’s battery level as a Birth-Death process.

At steady state and as shown in Figure 3, each IoT device has a battery level within interval $\left[\underline{B},\;\bar{B}\right]$. The battery level bounds, i.e., $\bar{B}$ and $\underline{B}$, must be defined according to the system application (data importance, sensing rate, etc.) and the battery type. Let denote λ the data rate of the IoT device and let μ represent the charging rate (i.e., amount of energy received) from the UAV station. The rate of charge varies according to the scheduling policy. Let $\prod_{k}$ be the probability that an IoT device is in a battery level *k*. After some algebra, we can write $\prod_{k}$ as follows:

(6) $$\prod_{k}=\frac{\mu_{k-1}\mu_{k-2}\ldots \mu_{1}}{\lambda_{k}\lambda_{k-1}\ldots \lambda_{1}\lambda_{0}}\cdot \prod_{0}=\prod_{l=0}^{k-1}\frac{\mu_{l}}{\lambda_{l+1}}\cdot \prod_{0},$$

where the charge rate of IoT device *i* is given by

(7) $$\mu_{k}\left(i\right)=\exp\left(-T_{nrg}^{i}\left(k\right)\right).$$

Furthermore, the average number of devices that are in a battery level *k* is given by:

(8) $$E\left[N_{k}\right]=\prod_{k}\cdot N_{N},$$

where *N* is the number of IoT devices covered by the UAV station in a given island N. Let *R* be the UAV coverage range and let η be the average number of IoT device per surface unit. Then, *N* can be estimated by:

(9) $$N=\eta \cdot \pi \cdot R^{2}.$$

**Figure 4:**
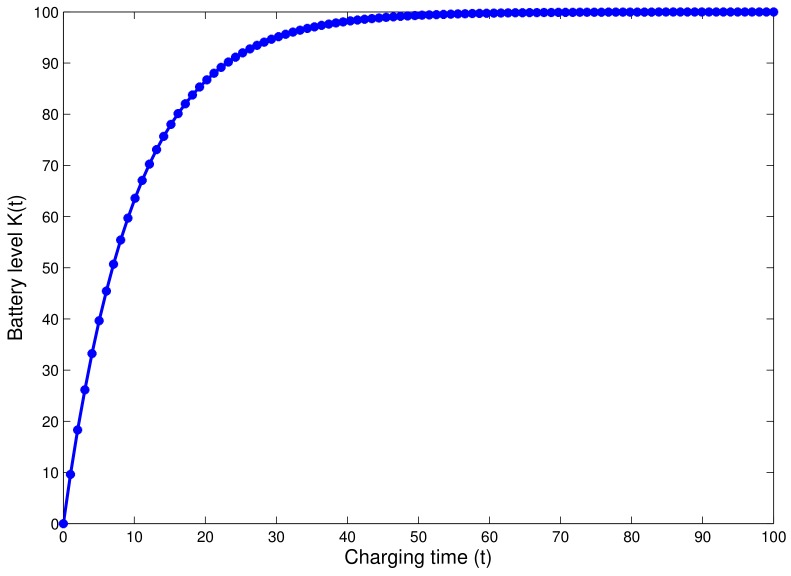
Battery recharging evolution over time.

When a ground IoT device receives energy from the UAV station, the device battery will be charged exponentially as shown in Figure 4. Thus, the battery level can be captured using the formula:

(10) $$K\left(t\right)=K_{max}\cdot \left(1-\exp\left(-\frac{t}{\tau }\right)\right),$$

where:

- τ is a time constant that expresses the battery charging rate as function of resistor impedance (R) and inductor (L) in which the battery is connected, thus $\tau =\frac{L}{R}$.
- $K_{max}$ is the maximum level that the battery can reach. Here, $K_{max}=B_{N}$.

## 3. Data Gathering and Energy Transfer Dilemma

Bringing the network closer (e.g., for data gathering or to deliver low-latency services) to end users is increasingly becoming a key function of wireless networks and also a hot topic. Here, we aim to use UAV to collect data from ground IoT devices. It is worth recalling that data gathering can be broadly classified into three categories: (1) The first scheme is “static data collection” in which the sink node is static, and the ordinary nodes upload data through multi-hop routing networks. The problem here is the high energy consumption by relay nodes that perform the routing between ordinary nodes and the sink, which results in a short network lifetime; (2) However, in the second method, the excessive energy consumption can be solved by using a mobile sink. In other words, this method uses vehicles, installed within a sink node, to collect data from the devices in the network. Despite this advantage of energy consumption, this kind of data collection is still limited, especially in environments where transportation is difficult; (3) The third approach consists of using aerial vehicles as flying sinks to collect data from the ground area. Now, the UAV plays a key role in data collection, due to its autonomous operations and unconstrained mobility, allowing for efficiently hovering over the monitored area (e.g., unreachable areas for human being). Furthermore, UAV collects data faster compared to other methods, as it has a higher movement speed and better radio conditions. This results in improving the network lifetime with highly scalable features for large scale networks. In addition, the UAV is characterized by a higher bandwidth, low latency and offers an extensive communication coverage.

The framework developed here could be very helpful in many realistic situations. Our main goal is to build a simple, efficient and scalable model for performance evaluation of UAV-empowered IoT and complex systems. For instance, it can be used in Ultra Dense Networks (UDNs), consisting of massive spatially distributed wireless nodes where batteries are the primary energy sources. In such a system, scalability is a key feature for efficient deployment. Now, our model will not only help with extending the network lifetime, but also has the advantage of being easy and mathematically tractable.

### 3.1. Ground IoT Device to UAV Link

We consider a set of *m* islands (remote areas), and each includes *N* IoT devices to be covered by the flying base station. The UAV retrieves information from islands in a sequential manner. It collects data from an island before moving to another island. The communication link between the IoT devices and the UAV depends on the UAV altitude. For instance, it can use a medium range standard such as IEEE 802.11 (WiFi), which allows a line-of-sight range up to several hundred meters and high data rates. However, if the UAV operates at a higher altitude spanning several kilometers, wide area networks (e.g., LoRaWAN, LTE, LTE-A, IEEE 802.16 (WiMAX)) would be preferred in this case as they can reach much higher data rates. In our model, we consider that the data to be collected by the UAV can be either best effort or time-sensitive traffic. Note that time delivery is critical when unusual events occur.

### 3.2. Data Collection Time and Battery Recharging Time

When hovering over a specific island, the UAV provides two services: (i) energy transfer, and (ii) data collection. During the first phase, the UAV wirelessly transfers energy to ground IoT devices with low battery levels. The second phase is dedicated to data collection from sensor devices with the aim to gather the maximum possible amount of data. Moreover, depending on the amount of data collected and coverage time allocated to an island, the energy transfer phase can be realized before and after data collection phase. It is worth mentioning that the limited energy of the UAV might influence its travel duration. Thus, to sustain both the efficiency of data collection and the network survivability by ensuring sufficient energy to depleted IoT devices, it is imperative to strategically determine the required time for each operation. Indeed, after the coordination between the UAV station and ground IoT devices during the joining stage where communication parameters are negotiated (see Figure 2), the UAV can estimate the required time for both data collection and batteries recharging. Then,

- the required time to collect data from an IoT devices of Island N is calculated by:
  (11) $$T_{data}^{N}=\sum_{i\in N}\frac{{DATA}_{i}}{\rho_{i}}\cdot 1_{\left\{B\left(i\right)>\underline{B}\right\}},$$
- the required time for energy transfer (batteries’ charging) is given by:
  (12) $$T_{nrg}^{N}=\sum_{k=B_{0}}^{\bar{B}}t_{nrg}^{k}\cdot E\left[N_{k}\right],$$

where

- $B\left(i\right)=B_{0},B_{1},\cdots ,B_{N}$ is the current battery level of IoT device *i*;
- $t_{nrg}^{k}$ is the allocated time to recharge a depleted ground devices of battery level *k*. This time allocation will be calculated for each IoT device and will be explained in the next subsection;
- $\bar{B}$ is a threshold beyond which no battery recharging is needed;
- $\underline{B}$ is the battery level’s critical threshold. At this battery status, an IoT device is forced to switch off its radio modules to keep alive;
- ${\mathrm{DATA}}_{i}$ is the data size to be transmitted by IoT device *i* to the UAV;
- $\rho_{i}$ is the traffic rate allocated to IoT device *i* by the hovering UAV;
- $1_{\left\{A\right\}}$ is an indicator function that equals 1 when expression *A* is true; it equals 0 otherwise.

Under homogeneous IoT devices, i.e., $DATA_{i}=DATA_{j}=DATA,\;\rho_{i}=\rho_{j}=\rho$, the data gathering time and the energy transfer time write respectively:

(13) $$T_{data}^{N}=N\cdot \frac{DATA}{\rho }\cdot \sum_{k=\underline{B}}^{B_{N}}\prod_{k},$$

and

(14) $$T_{nrg}^{N}=N\cdot \sum_{k=B_{0}}^{\bar{B}}t_{nrg}^{k}\cdot \prod_{k}.$$

## 4. Energy Transfer Policies

While hovering over an island and during the join phase, each ground IoT device sends its ID, its data rate demand, its current battery level, etc. Next, the UAV station seeks to schedule batteries (re)charging and data gathering. Thus, the UAV station needs to implement an efficient scheduling policy allowing to fairly share the energy among depleted ground devices and also to collect data from IoT devices over the window of coverage time reserved to a given island.

We improved network lifetime, and we include the battery level and the data priority of each device in defining the best scheduler. In other words, once the UAV station receives information about the batteries’ level from ground devices within the joining phase, it ranks them according to their respective battery level. Next, the UAV station decides the order of energy transfer and data gathering. Clearly, IoT devices with a critical battery level might need to be recharged first in order to be able transmit again their data. Alternatively, sensors with urgent data might also need to be served first. Henceforth, the UAV station is expected to meet an efficient energy-data trade-off, while facing a scheduling dilemma.

**Figure 5:**
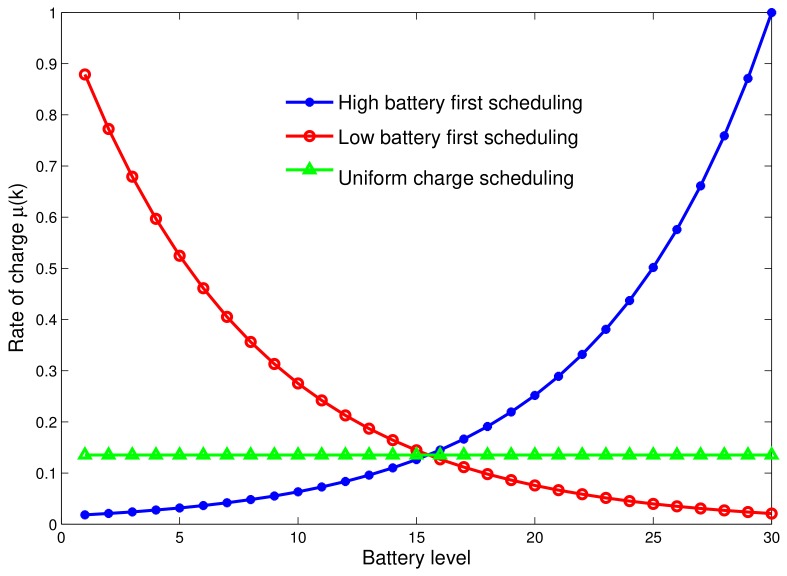
The rate of charge μ(k).

Let $T_{nrg}=\left(t_{nrg}^{1},t_{nrg}^{2},\cdots ,t_{nrg}^{N}\right)$ be the time vector required to recharge depleted ground IoT devices—of course, recharging time $t_{nrg}^{k}$ of IoT device *k*. It is worth noting that $T_{nrg}^{N}=<T_{nrg},\;1>$. We define three scheduling policies that could be considered according to the network activity profile/statistics. Of course, the average battery level over all IoT devices depends on the recharging time distribution. Figure 5 illustrates the charging rate against the actual battery level, for the three scheduling policies. This way, the recharge time allocated to an IoT device *i* whose battery level is *k* can be written as

(15) $$t_{nrg}^{i}\left(k\right)=\psi \left(k\right)\cdot T_{nrg},\;i=1,2,\cdots ,N\;\mathrm{and}\;B=B_{0},B_{1},\cdots ,B_{M},$$

where ψ(k) is the allocation function that depends on the scheduler used. Namely,

- Lowest Battery Level First (LBLF) scheduling: The UAV station first recharges out-of-battery IoT devices, then those with a battery level close to the critical threshold $\underline{B}$ and so on. Here, the recharging is asymmetrical as the UAV allocates strictly decreasing recharging time with battery status. This policy allows for keeping IoT devices alive and increasing the whole network lifetime. The recharging time per IoT device while being in state *k* is given by:
  (16) $$t_{nrg}^{i}\left(k\right)=T_{nrg}\cdot \frac{\left(\bar{B}-k\right)\cdot 1_{\left\{k<\bar{B}\right\}}}{\sum_{i\in N}\left[\bar{B}-B\left(i\right)\right]\cdot 1_{\left\{B\left(i\right)<\bar{B}\right\}}},\;i=1,2,\cdots ,N\;\mathrm{and}\;B=B_{0},B_{1},\cdots ,B_{M}.$$

- Highest Battery Level First (HBLF) scheduling: During the energy transfer phase, the UAV station starts recharging devices with battery level close to threshold $\bar{B}$. Here, the allocated time is increasing with the battery level. Using this scheme within a network with unbalanced battery levels is likely to decrease the network lifetime. The HBLF scheduling function can be expressed as follows:
  (17) $$t_{nrg}^{i}\left(k\right)=T_{nrg}\cdot \frac{k\cdot 1_{\left\{k<\bar{B}\right\}}}{\sum_{i\in N}B\left(i\right)\cdot 1_{\left\{B\left(i\right)<\bar{B}\right\}}},\;i=1,2,\cdots ,N\;\mathrm{and}\;B=B_{0},B_{1},\cdots ,B_{M}.$$

- Uniform Recharging (UR) scheduling: All depleted devices receive the same amount of energy from the UAV station. This scheme has some good fairness properties but might fail in extending the network lifetime. The UR scheduling function is written as follows:
  (18) $$t_{nrg}^{i}\left(k\right)=T_{nrg}\cdot \frac{1}{\sum_{i\in N}1_{\left\{B\left(i\right)<\bar{B}\right\}}},\;i=1,2,\cdots ,N\;\mathrm{and}\;B=B_{0},B_{1},\cdots ,B_{M}.$$

**Figure 6:**
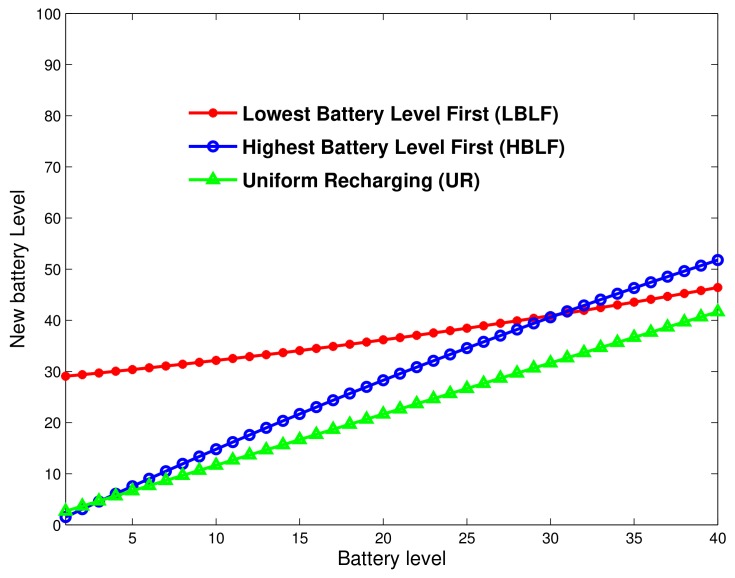
Scheduling policy.

Figure 6 shows an example of how to perform each recharging scheme. We plot here the average battery level before energy transfer and after the recharge is performed. We note that LBLF performs well for low battery levels as the scheduler starts first by recharging the low battery. One notices that the uniform scheduler performs very badly as it definitely leads to a decreased network lifetime.

### UAV Energy Balance: Consumption V.S. Harvesting

After the coordination/join phase, the UAV charges depleted devices that have a battery level below the threshold $\bar{B}$. In addition, we distinguish two categories of the devices classified with a low battery level. The first one includes devices capable of sensing and transmitting information even if their batteries are below the threshold. The second category comprises devices that are close to battery exhaustion, i.e., whose battery level is below the critical threshold $\underline{B}$. Those ground devices have to be prioritized and will be recharged first by the UAV station. Here, we are interested in investigating both energy consumed and energy harvested by the UAV station itself.

The main energy consumption (energy consumed by mechanical modules is not considered) of the UAV is due to its radio operation. Let $P_{uav,\;i}$ be the transmit power used by the UAV to transfer energy to IoT device *i*. We recall that only IoT devices whose battery levels are below $\bar{B}$ would be recharged. The energy consumed by the UAV while transferring energy to low-battery devices is:

(19) $$E_{cons}^{uav}=\sum_{i\in N}P_{uav,\;i}\cdot t_{nrg}^{i}\left(k\right)\cdot 1_{\left\{B\left(i\right)<\bar{B}\right\}}.$$

Under homogeneous IoT devices, Equation (19) writes:

(20) $$E_{cons}^{uav}=N\cdot P_{uav}\cdot \sum_{k=B_{0}}^{\bar{B}}t_{nrg}\left(k\right)\cdot \prod_{k}.$$

Once energy transfer phase is over, the UAV station switches to reception mode in order to gather data from ground devices. Now, each ground device *i* sends its sensed data at transmit power $P_{i,\;uav}$ to the UAV station. According to the join phase, the UAV station computes the transmission time $T_{data}^{i}$ to be allocated to each IoT device *i*. While equipped with an energy harvesting module, the flying UAV can extract energy from RF signals transmitted by ground devices. Clearly, energy harvested from ground devices’ signals could be ignored as it is very small and depends on the harvesting gain, transmit power and the radio channel state. In addition, the UAV can carry an external energy source, such as a photovoltaic module, allowing high survivability. Without loss of generality, the harvested energy is strongly related to the transmit power of ground devices and the collected data volume. For network lifetime purposes, only IoT devices whose battery level is higher than $\underline{B}$ could transmit data. The amount of energy harvested by the UAV station can be easily estimated by:

(21) $$E_{harv}^{uav}=\sum_{i\in N}P_{i,\;uav}\cdot H_{i,uav}\cdot t_{data}^{i}\cdot 1_{\left\{B\left(i\right)>\underline{B}\right\}},$$

where $H_{i,uav}$ is the average channel gain between the IoT device *i* and the hovering UAV. Notice that $H_{i,uav}=\int_{0}^{\infty }H_{i}\cdot \Psi \left(H\right)dH$, where $H_{i}$ is the instantaneous channel experienced by IoT device *i* and Ψ(·) is the channel distribution (Rayleigh, Path-loss, etc.). Here, the channel gain depends mainly on the distance between *i* and the UAV as areal communications usually experience the Non-line-of-Sight (NLOS) mode. Under homogeneous IoT devices with $P_{i,\;uav}=P_{j,\;uav}=P$, Equation (21) writes:

(22) $$E_{harv}^{uav}=N\cdot P\cdot {\tilde{H}}_{uav}\cdot \sum_{k=\underline{B}}^{\bar{B}}t_{data}\left(k\right)\cdot \prod_{k},$$

where ${\tilde{H}}_{uav}=\frac{1}{N}\sum_{i\in N}H_{i,uav}$ is the average channel experienced by all IoT devices. The UAV problem is now to compute the optimal value of α allowing to meet an efficient trade-off. Namely,

(23) $$\alpha^{*}=\underset{\alpha }{arg max}\;\alpha \cdot E_{cons}^{uav}+\left(1-\alpha \right)E_{harv}^{uav}.$$

## 5. Traveling Salesman Problem with Time Windows: Shortest Traveling Time Trajectory (STTT)

Along this article, we deal with the problem of energy efficiency in UAV-assisted IoT environments. We mainly dealt with defining an efficient trade-off between data gathering and energy transferring. However, we did consider the energy consumed by the trajectory and the order to serve IoT islands. Thus, it is important to seek an energy efficient path that should be followed by UAV in order to fly over all IoT islands while spending a minimum amount of energy. For this, we consider a network formed by *m* islands/clusters. We assume that the distances between each pair of clusters are known. A m∗m time-dependent matrix $T=\{t_{i,\;j}\}$ appends the duration (temporal cost) between each pair of clusters (i,j)—in addition to each cluster possibly having its own time window $\left[s_{i},\;e_{i}\right]$, in which the UAV covers the cluster, and where $s_{i}$ and $e_{i}$ indicate the time of the first and the last service respectively presented in a covered cluster. Of course, the stop-over location of the UAV while serving a cluster is a key parameter to set. Hence, the distance between two stop-overs is given as follows:

(24) $$q_{j,\;j^{\prime }}=\sqrt{{\left(x_{j}-x_{j^{\prime }}\right)}^{2}+{\left(y_{j}-y_{j^{\prime }}\right)}^{2}},\;\;\;j,j^{\prime }=1,2,\cdots ,N.$$

According to [29], the energy consumption when a UAV is moving between two stop locations can be obtained by the following expression:

(25) $$E_{j,\;j^{\prime }}=\int_{0}^{\frac{q_{j,\;j^{\prime }}}{v}}P_{j,\;j^{\prime }}\;dt\;=\;\frac{q_{j,\;j^{\prime }}}{v}\cdot P_{j,\;j^{\prime }},\;\;\;j,j^{\prime }=1,2,\cdots ,N,$$

where $P_{j,\;j^{\prime }}$ is the power consumed by the UAV’s movement. Then, the time spent between two clusters *j* and $j^{\prime }$ writes:

(26) $$t_{j,\;j^{\prime }}=\frac{E_{j,\;j^{\prime }}}{P_{j,\;j^{\prime }}},\;\;\;j,j^{\prime }=1,2,\cdots ,N.$$

The UAV’s problem is then to find the shortest trajectory achieving the minimum delay to collect data and to transfer energy to ground IoT devices. The UAV has to visit once all IoT islands and return to its starting location assumed to allow for transferring sensed data to a sink or a processing unit. Here, we need to solve the traveling salesman problem, considering the temporal cost matrix $T_{j,\;j^{\prime }}$ as a cost function. In summary, the UAV adopts Algorithm 1 to seek an optimum trajectory at a minimum travel time.
**Algorithm 1** Shortest Traveling Time Trajectory (STTT)
1:***Input:** M*, $E_{j,\;j^{\prime }}$ and $P_{j,\;j^{\prime }}$2:***Output:*** Trajectory with a minimum time.3:Compute the matrix $\left[t_{i,j}\right]=T_{j,\;j^{\prime }}+T_{cover}^{j^{\prime }}$ of UAV travel time..4:**Row minimization**5:**for** Each *i* **do**6:  **for** Each i≠j **do**7:    ***Select*** $k_{i,j}=\min_{j\in M}\left[t_{i,j}\right]$8:    **for** Each *j* **do**9:      $\left[t_{i,j}\right]-k_{i,j}$10:      ***Column minimization***11:      **for** Each *j* **do**12:        **for** Each j≠i **do**13:          ***Select*** $k_{ij}=\min_{i\in M}\left[t_{i,j}\right]$14:          **for** Each *i* **do**15:            $\left[t_{i,j}\right]-k_{i,j}$16:        **for** Each *i* **do**17:          ***Compute of penalty***18:          **for** Each $t_{i,j}=0$ **do**19:            ***Select*** $\min_{i\in M}\left[t_{i,j}\right]=K$20:            ***Select*** $\min_{j\in M}\left[t_{i,j}\right]=L$21:            ***And*** $[t_{i,j}]=K+L$22: Struck off the row and column corresponding to 0 with maximum penalty, and make assignment from i to j.23: Reduced matrix: Rewrite the matrix without consider the row and column corresponding to 0 with maximum penalty.24:**return:** Set of assignment from i to j 25:**End.**


## 6. Simulations and Numerical Investigations

In this section, we implement the proposed model with MATLAB software to evaluate the performance and the added value of using UAV-based flying stations with energy transfer capabilities and energy harvesting modules. To this end, we study an example of a network composed of 1000 IoT devices distributed following a PPP in a geographical area. We consider a single UAV station serving ground IoT devices. Table 1 summarizes the main values of the parameters used for numerical examples and simulations.

We analyze the behaviors of the energy harvested by the UAV station and on the energy transferred to IoT devices as functions of both data gathering time (i.e., during which UAV station harvests energy) and devices recharging time. As mentioned earlier, the UAV coverage time is split into two parts weighted by α; energy transfer time $T_{nrg}=\alpha \cdot T_{cover}$, and data gathering time $T_{data}=\left(1-\alpha \right)\cdot T_{cover}$. We aim here to find out an efficient trade-off to ensure high network lifetime (thus better energy efficiency) and satisfactory data gathering performance.

### 6.1. Effect of Data–Energy Tradeoff Parameter α

**Figure 7:**
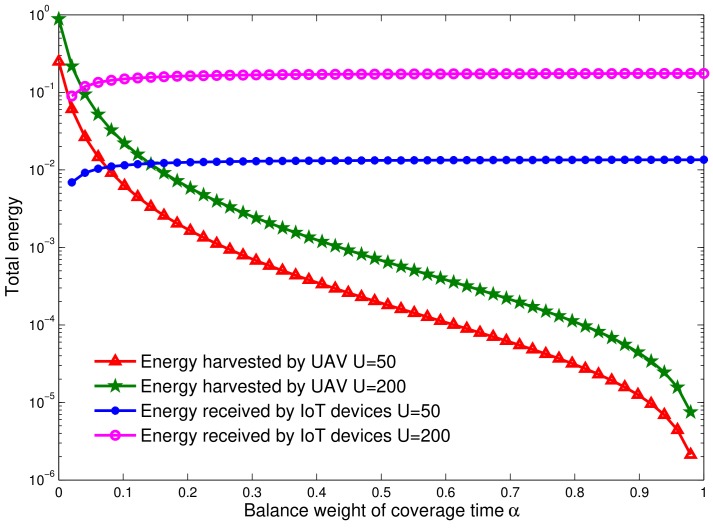
Total energy harvested by UAV and received by IoT devices.

We depict in Figure 7 the energy harvested by the UAV station and the energy transferred to ground IoT devices as functions of the energy transfer/harvesting trade-off parameter, for both 50 IoT devices and 200 IoT devices. We notice that the energy received by IoT devices is increasing as the value of α increases. However, this behaviour is quite intuitive since increasing α allows for allocating more recharging time to the UAV station to serve depleted IoT devices. However, this comes to the detriment of the energy harvesting time. Meantime, the amount of energy harvested by the UAV decreases as the data transmit period becomes smaller, which implies less energy harvesting opportunities. In order to meet an efficient trade-off, one can solve Equation (23). Alternatively, the UAV operator can optimize the network lifetime (equivalently, the average battery level for ground devices) while guaranteeing a target data throughput. Furthermore, the operator can seek to optimize the data rate while ensuring a target network lifetime (i.e., a target battery level for IoT devices).

Figure 7 also shows the interplay of α on harvested energy and transferred energy in terms of the network density. We observe that the denser the network, the greater the harvested energy by the UAV station and the greater the average battery level of IoT devices.

### 6.2. Effect of Data Transmission Rate by IoT Devices

**Figure 8:**
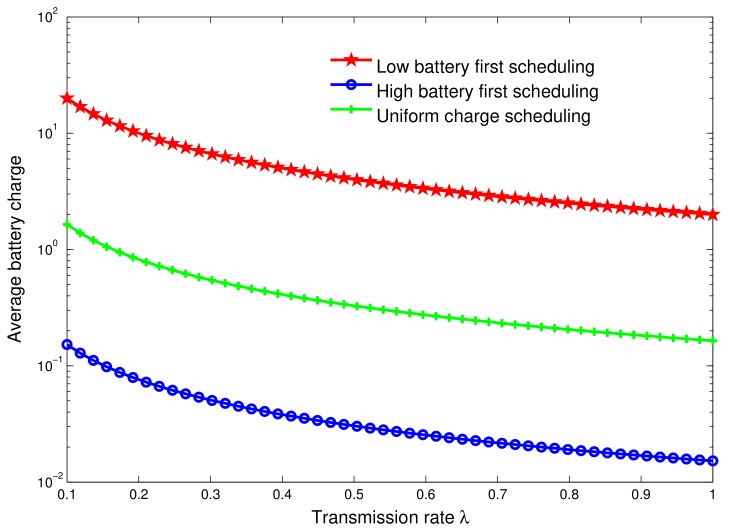
The average battery charge.

Next, we plot in Figure 8 the average battery charge as a function of the IoT device transmission rate, λ∈[0,1], for the three energy transferring policies defined earlier. Notice that the transmission rate of an IoT device depends on the data sensing frequency that defines the data freshness. This mainly depends on the parameter the sensor is monitoring. We remark that the average battery charge decreases as the value of transmission rate increases, which is quite intuitive. Indeed, increasing the rate of transmission increases the energy consumed by IoT devices, so that the data gathering time is higher than the recharging time. Consequently, this results in a higher number of depleted devices that might require to be recharged shortly. When the system is more interested in data gathering (e.g., in case of an environment change being detected) than increasing devices’ lifetime, the UAV station ignores the battery level of devices and allocates a shorter recharging time. This is why the average battery charge decreases for all schemes. More specifically, the average battery charge, in the case of low battery first scheduling, is more efficient compared to others’ policies, as it yields higher average battery level. This can be explained by the fact that the energy given by the UAV station is shared fairly between depleted devices, as the UAV counts for the battery level of each device to extend the network lifetime.

### 6.3. Energy Harvesting Gain

In order to capture the added-value of our scheme, we define the energy harvesting gain as the ratio between the energy level before and after the energy harvesting operation. Namely,

(27) $$G=\frac{E_{\mathrm{new}}}{E_{\mathrm{old}}},$$

where

- $E_{\mathrm{old}}:$ is the energy level of the UAV station when arriving to the island;
- $E_{\mathrm{new}}:$ is the UAV current energy budget, i.e., after performing both operations of data collection and energy transfer from/to IoT devices (see Equations (19) and (21)). It is given by
  (28) $$E_{\mathrm{new}}=E_{old}+E_{harv}^{uav}-E_{cons}^{uav}.$$

**Figure 9:**
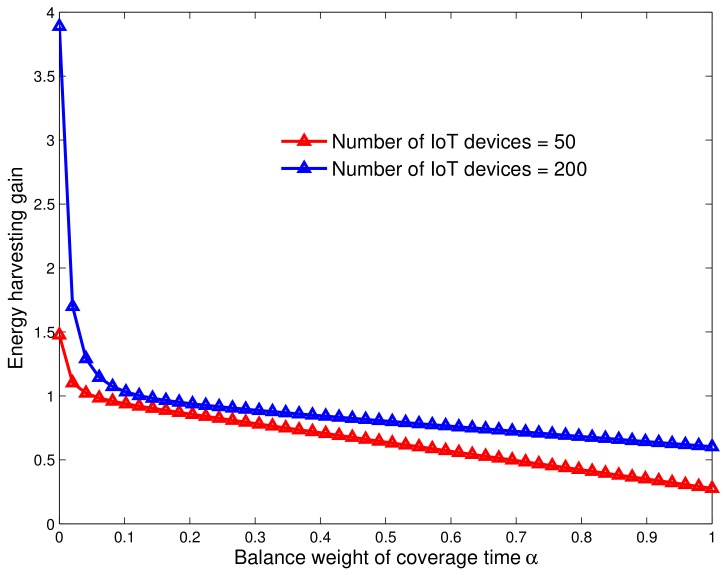
Energy harvesting gain versus α.

As mentioned earlier, the main idea behind using energy harvesting module is to solve the problem of limited battery for UAV and IoT environments. We plot in Figure 9 the energy harvesting gain (see Equation (27)) as a function of the trade-off coefficient α for two different network densities (50 and 200 IoT devices). From the figure, we observe that the gain is higher under low values of α. This is because the time allocated to data collection is higher with small values of α. This is because the time allocated to data collection is higher with small values of α, and consequently the amount of energy harvested is higher too. However, in the reverse case, the recharging time gets longer because α increases and the energy of UAV station is decreasing fast. Thus, the gain decreases. It is worth noticing that, regardless of the parameter of weight balance, the gain is more significant for dense networks, as the UAV station gives more coverage time to a given island.

**Figure 10:**
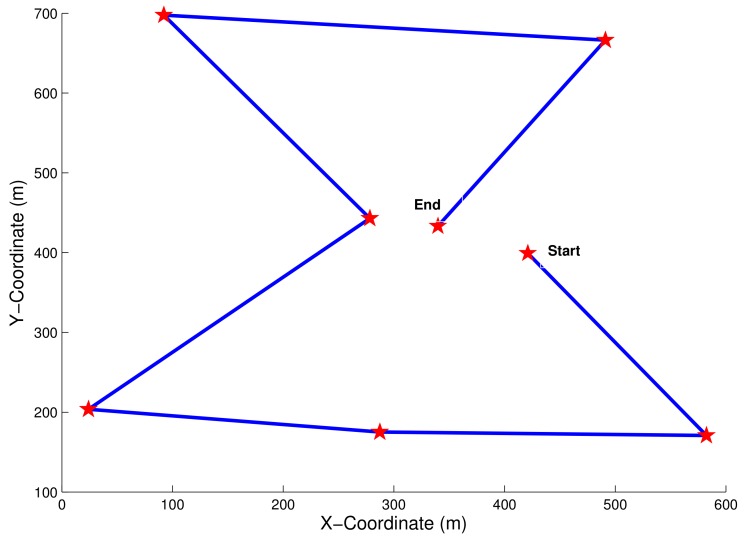
UAV trajectory in one travel.

Now, we turn to evaluate the optimal trajectory to be followed by a UAV while serving several IoT islands. In Figure 10, the red stars represent the stop locations of UAV at each island to guarantee an efficient uplink data transmission and downlink energy recharging. In order for the UAV station to spend a minimum total energy to fly over all these islands, and, compared to the random UAV trajectory, it is worth adopting the STTT algorithm (see Algorithm 1) that leads to an optimal trajectory as mentioned. At each stop-over, the UAV station takes into account the time between islands and the coverage time to be allocated for each island, which depends on the island density, in order to remain operational for a long time and achieve henceforth an efficient energy transfer/data collection trade-off.

### 6.4. Discussion

We recall that the aim of this work is to meet a high network lifetime while granting a satisfactory network access for ground IoT devices. First, we analyze the network average battery level under three energy transferring policies. Namely, we consider the low battery first scheme, high battery first scheme and uniform charging scheme. According to simulation results, we conclude that meeting a high network lifetime requires adopting a low battery first scheme. Otherwise, depleted devices would die and then drastically reduce the network lifetime, which is not desirable. Second, we established a data collection and energy transfer trade-off, by fine-tuning the weight α, where a flying network provider would achieve a good network availability while the ground network is still operating longer. Third, we analyze the added value (as energy harvesting) gain brought by the energy harvesting module under the trade-off coefficient α, in which the UAV operator can allocate more time to data collection with a smaller value of α depending on its interest. One notices that the gain is more important for dense and ultra-dense networks. Finally, we propose an optimal trajectory allowing for extending the network lifetime as long as possible. The optimal trajectory to be followed by UAV stations to serve several IoT islands is obtained using the shortest traveling time trajectory algorithm instead of taking a random trajectory. Of course, this optimal trajectory is only feasible for services with no quality of service requirements or for best-effort-like services. A service-aware version could be derived considering the quality of service constraints.

## 7. Conclusions

In this article, we propose a novel use case for UAVs wherein they are jointly used as flying base stations and as an energy source for depleted IoT devices. This scheme allows for building a flying/nomadic infrastructure to gather data sensed by ground IoT devices and at the same time allows for extending the network lifetime by transferring energy to out-of-battery devices. Both functions are performed during the coverage time over a given island composed of several IoT devices and sensors. Of course, the coverage time of an UAV is limited by the UAV autonomy. Here, we aim to define an efficient trade-off between data gathering time and ground devices’ batteries recharging time. We model the battery level of an IoT device as a Birth-Death process depending on the device activity rate (data sensing, data transmission, etc.) and on the amount of time it is getting recharged by the UAV. Furthermore, we investigate the optimal trajectory of UAV to fly over several islands and to perform both operations mentioned above while keeping an efficient data-collection/energy-delivery trade-off. Extensive numerical examples and simulations results show that our scheme is offering good performance and define an efficient data-gathering/energy-delivery trade-off under different battery recharging scheduling policies.

As a future work, we are seeking to optimize the IoT network lifetime by allowing some devices to strategically switch to sleep mode while there is no important data to sense or there are other neighboring IoT devices who might sense the similar data in terms of quality and freshness.

## Figures and Tables

**Table 1:** Simulation settings.

Parameter	Value
Maximum number of devices within a single island	200
Maximum transmit power of a ground IoT device	500 mW
Maximum battery level	100%
Threshold battery level	$\bar{B}=40\%$
Critical threshold	$\underline{B}=10\%$
Channel gain	Rayleigh with mean 1
Path loss exponent	3
